# The association between chronotype and incident dementia: exploring age, educational-attainment and sex differences

**DOI:** 10.1017/S2045796026100687

**Published:** 2026-05-07

**Authors:** Ana Neeltje Wenzler, Aart C. Liefbroer, Richard Oude Voshaar, Nynke Smidt

**Affiliations:** 1 Department of Epidemiology, University of Groningen, University Medical Center Groningen, Groningen, The Netherlands; 2 Netherlands Interdisciplinary Demographic Institute (NIDI)–Royal Netherlands Academy of Sciences (KNAW), The Hague, The Netherlands; 3 Department of Sociology, Vrije Universiteit Amsterdam (VU), Amsterdam, The Netherlands; 4 Department of Psychiatry, University of Groningen, University Medical Center Groningen, Groningen, The Netherlands

**Keywords:** biological rhythms, chronotype, cohort study, dementia, elderly, survival analysis

## Abstract

**Aims:**

Chronotype reflects individual variation in circadian rhythm (CR) (e.g., morningness versus eveningness). CR and chronotype have been associated with dementia pathology, and recent literature suggests that impaired sleep quality and CR disturbances may represent relevant causal factors for dementia. Current evidence is scarce and consists of just a small number of cross-sectional analyses and one longitudinal study. The aim of this study is to investigate the longitudinal association between chronotype and dementia risk in the older adult population, aged 60 years and older.

**Methods:**

Linking data from the Lifelines Cohort Study and data from Vektis obtained by Statistics Netherlands led to a sample for analysis of *n* = 16,757 participants. Chronotype was measured with the Munich ChronoType Questionnaire (MCTQ) between 2011 and 2015. Chronotype was categorised into five categories: extremely early, slightly early, intermediate, slightly late, extremely late. Dementia incidence was based on having at least one dementia indicator from the Vektis dataset in the years 2018 until 2024. A discrete-time survival model was used to examine the association between chronotype and dementia. Age was specified as the underlying time scale in the discrete-time logistic survival analysis. Sex was included as a covariate. Interactions between age, sex, and educational attainment and chronotype were measured by including relevant interaction terms in the model.

**Results:**

The median age in the sample was 65 years old, and 54% was female. In total, 7% got dementia during a mean follow-up period of 6.6 years. Most individuals had an intermediate chronotype (56%), with about 20% of the individuals having a slightly early and late chronotype, and only about 3% having an extremely early and late chronotype. Individuals with a slightly early (HR, 1.26 [95% CI: 1.08, 1.46]) and an extremely late chronotype (HR, 1.42 [95% CI: 1.00, 2.02]) had an elevated risk of dementia. There were no significant interactions.

**Conclusions:**

Having a slightly early as well as an extremely late chronotype was associated with an elevated risk of dementia.

## Introduction

Population ageing has contributed to a rise in dementia cases, with an estimated 55 million people affected globally; a number projected to grow to 153 million by 2050. According to the Lancet Commission, approximately 45% of dementia cases are attributable to 12 modifiable risk factors (Livingston *et al.*, 2024). Sleep disturbances are not included, as evidence remains inconclusive. The Commission proposes impaired sleep quality and circadian rhythm (CR) disturbances as potential causal factors (Livingston *et al.*, 2024). The CR – a 24-hour cycle regulating processes such as sleep (Chauhan *et al.*, 2023) – is reflected in chronotype, often called an ‘early lark’ or a ‘night owl’. Chronotype is partially influenced by age, with individuals typically shifting to a later chronotype during adolescence, followed by a gradual return to an earlier chronotype with increasing age (Fischer *et al.*, 2017). By midlife, chronotype stabilises, though 19.9% remain evening types and 7.1% adapt extreme morning types (Roenneberg *et al.*, 2019). Dim light melatonin onset is accurate but rarely used in large studies due to time, cost and participant burden (Kantermann *et al.*, 2015; Roenneberg *et al.*, 2019). As an alternative, self-report questionnaires such as the Munich ChronoType Questionnaire (MCTQ) are commonly used (Roenenberg *et al.*, 2015; Chauhan *et al.*, 2023). The MCTQ determines chronotype using sleep and wake times on workdays and free days by calculating Mid-point Sleep on Free days corrected for sleep debt on workdays (MSF_SC_) (Roenneberg *et al.*, 2007). Midpoint sleep time is a precise behavioural indicator of circadian phase and has been validated against biomarkers like DLMO (Kantermann *et al.*, 2015). The MSF_SC_ outcome from the MCTQ provides a continuous chronotype measure, ranging from extremely early (<12:00 am) to extremely late chronotypes (>9:00 am) (Juda, 2010).

The pathways through which chronotype may influence dementia risk are not fully understood. Chronotype reflects individual variation in a CR, such as morningness versus eveningness. A disrupted CR has been associated with dementia pathology (Musiek *et al.*, 2015). Neurodegeneration within the suprachiasmatic nucleus (SCN) can disrupt circadian regulation (Musiek and Holtzman, 2016). Conversely, CR disruptions may impair memory and cognitive functioning through SCN neurodegeneration (Montaruli *et al.*, 2021), enhanced amyloid-β accumulation (C. Wang and Holtzman, 2020) and increased oxidative stress from free radicals and reactive oxygen species (Wilking *et al.*, 2013), all linked to elevated dementia risk. This suggests a bidirectional relationship between chronotype and dementia. CR disruption may arise from shift work (Boivin *et al.*, 2022) or repeated jet lag (Vosko *et al.*, 2010), thereby contributing to neurodegeneration (Moeller and Kriegsfeld, 2023). Several studies have shown that a late chronotype is associated with negative health outcomes, independent of sleep debt or social jetlag (Didikoglu *et al.*, 2019; Partonen, 2023). Recently, variations in chronotype have been linked to cognitive functioning (Thapa *et al.*, 2020; Moeller and Kriegsfeld, 2023), cognitive decline (Wenzler *et al.*, 2025) and dementia (Guo *et al.*, 2024). The only longitudinal study had respondents categorised as either early or late chronotype, without acknowledging intermediate chronotypes (Guo *et al.*, 2024). Additionally, the direction of the results was unclear. Many studies have relied on the Morningness-Eveningness Questionnaire (MEQ), which assesses a personality trait rather than the phase of entrainment (i.e., the timing of your biological clock) (Roenneberg, 2015). The limited evidence on this topic, combined with the Lancet Commission’s call for more research (Livingston *et al.*, 2024), highlights the importance of examining chronotype as a potential risk factor for dementia.

The primary aim of this study is to investigate the association between chronotype and dementia among older adults in the general population. We hypothesise that a late chronotype is associated with an increased risk of dementia. Given the evidence that dementia risk is higher among women and individuals with lower educational attainment, and increases with age, moderation by age, sex and educational attainment will be examined.

## Methods

### Study design and population

For this study, we linked national register data on dementia healthcare claims to the Lifelines cohort study. Lifelines is a multi-disciplinary prospective population-based cohort study examining in a unique three-generation design the health and health-related behaviours of 167,729 persons living in the North of the Netherlands. It employs a broad range of investigative procedures in assessing the biomedical, socio-demographic, behavioural, physical and psychological factors which contribute to the health and disease of the general population, with a special focus on multi-morbidity and complex genetics, as extensively described elsewhere (Scholtens *et al.*, 2015). All included participants signed informed consent. Dementia diagnoses were identified using healthcare claims from Vektis, a registry of all healthcare claims in the Netherlands. These data, along with mortality information, are available within the System of Social Statistical Databases (SSB) of Statistics Netherlands. Lifelines participants were securely linked to the SSB by Statistics Netherlands, enabling the integration of dementia data within Lifelines. All analyses were conducted within this secure environment. A study timeline is provided in Figure S1 Supplementary Material.

**Figure 1.**
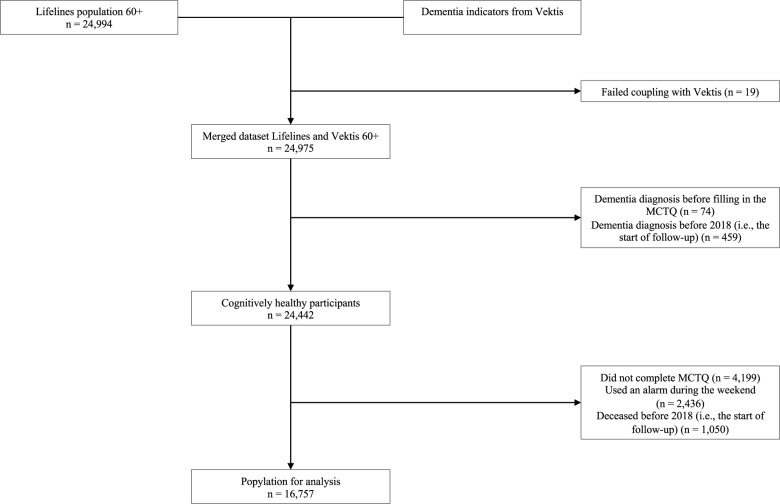
Flowchart of study participants.

All Lifelines participants aged ≥60 years (*n* = 24,994) were linked to CBS data; after excluding 19 mismatches, 24,975 remained. Next, we excluded all participants who had developed dementia before 2018, based on information in Lifelines and data obtained from Vektis before 2018 (*n* = 533). This resulted in a cognitively healthy study sample of 24,442 participants. Participants without chronotype data (missing MCTQ, *n* = 4,199 or weekend alarm use, *n* = 2,436) or who died before 2018 (*n* = 1,050) were excluded, yielding a final sample of 16,757 (Fig. 1).

### Measurements

#### Dementia

Incident dementia defined using healthcare claims from Vektis, including (1) dementia-related medication, (2) dementia-related visits to neurology, geriatric or internal medicine clinics, (3) dementia-related mental healthcare, (4) community nursing for dementia and (5) Long-term Care Act (WLZ) eligibility assessments for dementia. The availability and completeness of these indicators have improved over time; since 2017, their combined use provides a realistic representation of dementia cases (van den Pol, 2020) (Table S1 Supplementary Material), giving the opportunity to calculate the incidence of dementia from 2018 onwards. Although informative, these claims serve as an indirect proxy for dementia. Dementia was modelled as a time-varying dummy variable, coded 1 in the year the participant had ≥1 indicator and 0 in all preceding years.

#### Chronotype

Chronotype was assessed at baseline with the Dutch version of the MCTQ (Zavada *et al.*, 2005). Chronotype was defined as the MSF_SC_. Adjusting for sleep debt avoids misclassifying individuals as having a stronger evening preference then they actually do (Roenneberg *et al.*, 2003). Each 1-hour increase in mid-point of sleep reflects a 1-hour shift in chronotype, ranging from extremely early to extremely late. Participants who reported using an alarm clock during the weekend were excluded, as the MSF_SC_ requires natural waking times. Because category cut-offs for the MSF_SC_ can be arbitrary, chronotype was included as a continuous variable (Juda, 2010). For categorical analysis, the following groups based on MSF_sc_ cut-offs were used: extremely early (≤1:30), moderately early (>1:30 and ≤2:30), slightly early (>2:30 and ≤3:30), intermediate (>3:30 and ≤4:30), slightly late (>4:30 and 5:30), moderately late (>5:30 and ≤6:30) and extremely late (>6:30) (Roenneberg *et al.*, 2019). Due to the small subgroup sizes, the extremely early (*n* = 57) and extremely late (*n* = 73) groups were combined with the moderately early or moderately late chronotype, respectively.

#### Confounders

Age was used as the underlying time scale in the discrete-time logistic survival analysis. Age at baseline and age at the end of follow-up were included, the latter defined as age at dementia diagnosis, death, age 95 years or last observation. Sex was included as a binary variable, female or male. Educational attainment was assessed with the question ‘What is the highest level of education you have completed?’ and categorised in three groups: low (junior general secondary education or lower), middle (secondary vocational education, work-based learning or higher general secondary education) and high (higher vocational education or university) educational attainment.

### Statistical analysis

Descriptive statistics were calculated to describe baseline characteristics. Age was presented by median and interquartile range (IQR) because the variable was non-normally distributed. Sex, educational attainment and chronotype categories were presented as number and percentages (*n*, %).

A discrete-time survival model was estimated to examine the association between chronotype and dementia. The analysis was right censored at 95 years old, as the sample size above this age was too small to extract reliable estimates. Model 1 assessed the underlying hazard of age on dementia risk, including a quadratic term to account for potential non-linearity. Model 2 examined the linear association between chronotype and dementia risk, additionally adjusting for sex. If no linear association was observed, a spline model was used to explore non-linearity. When non-linearity was indicated, chronotype was categorised. Exact *p*-values were presented, with a *p* < 0.05 deemed statistically significant. Effect modification by age, sex and educational attainment was assessed using log-likelihood ratio tests (LRT) comparing models with and without the interaction terms. Because the MCTQ was administered between 2011 and 2015, the time to the first possible dementia diagnosis in 2018 varied across participants. This interval, referred to as lag time, was included as a covariate in all analysis (Figure 1, Supplementary Material).

As a sensitivity analysis, chronotype was re-categorised into three groups: early (≤3:30), intermediate (>3:30 & ≤4:30) and late (>4:30), to provide a simplified representation of its association with dementia risk. The model used the intermediate chronotype as the reference and was adjusted for sex and lag time. In a second sensitivity analysis, there was examined whether sleep duration explained the association between chronotype and dementia. Sleep duration was included as a continuous variable, measured in hours using the MCTQ.

Statistical analyses were performed in R, version 4.3.1. The discSurv package was used for the discrete time analysis.

## Results

Table 1 presents the characteristics of the study sample (*n* = 16,757). Participants had a median age of 65 years (IQR, 62–69), and 54% of whom were women. The mean follow-up time was 6.6 years, during which 7% developed dementia and 9% died. Most participants had an intermediate chronotype (56%), followed by slightly early (20%) and slightly late (19%) chronotypes. Extremely early (2%) and late (3%) chronotypes were rare. Over half of the participants had low educational attainment (51%) while the middle and high groups were equally represented (23%).

**Table 1.** Population characteristics

Variables	Population for analysis *n* = 16,757
Age (median [IQR])	65 (62–69)
Sex – female (*n* [%])	9,093 (54%)
Follow-up years (mean [SD])	6.6 (1.2)
Dementia indication (*n* [%])	1,088 (7%)
Deceased (*n* [%])	1,437(9%)
Chronotype (*n* [%])	
Extremely early	345 (2%)
Slightly early	3,319 (20%)
Intermediate	9,434 (56%)
Slightly late	3,210 (19%)
Extremely late	449 (3%)
Educational attainment (*n* [%])	
Low	8,582 (51%)
Middle	3,755 (23%)
High	3,894 (23%)
Missing	526 (3%)

**Figure 2.**
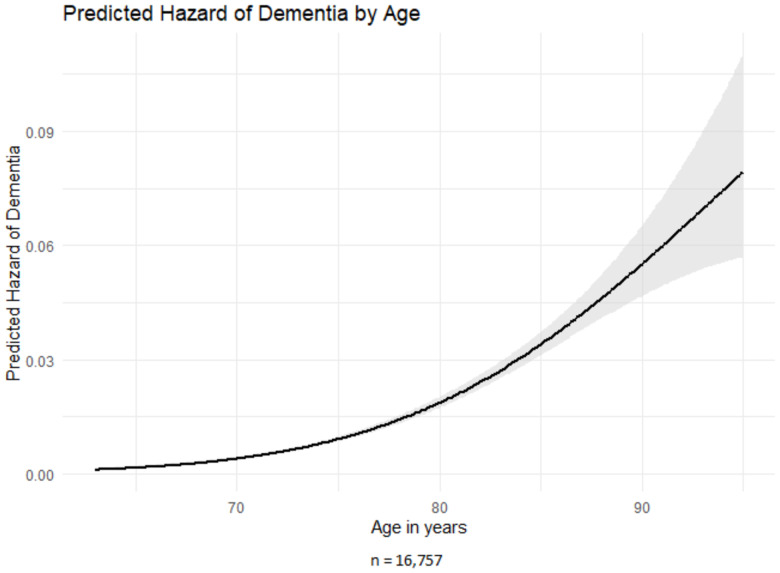
Predicted hazard of dementia by age.

Model 1 assessed whether age was associated with dementia risk and showed a curve-linear relationship (Fig. 2). Dementia risk increased with age, while the small but statistically significant quadratic term indicated a slight decrease in hazard over time. Lag time, ranging from 3 to 7 years, had no effect on this association (Table S2 Supplementary Material).

**Figure 3.**
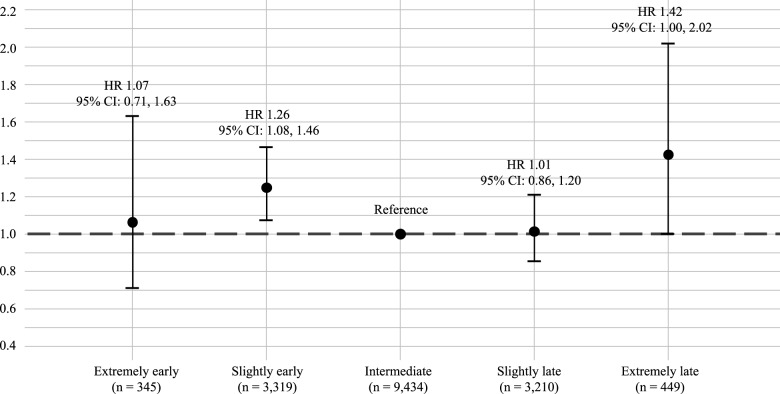
Risk of dementia (hazard ratio [HR]) by chronotype during average 6.6 years of follow-up.

Model 2 examined the association between chronotype and dementia risk. No linear association was found between continuous chronotype (MSF_SC_) and dementia (HR, 0.95 [95% CI: 0.88, 1.03]) (Table S3, Supplementary Material). Sex and lag time were not associated with dementia risk. The distribution of the MSF_SC_ is presented in Figure S2 Supplementary Material. A spline model suggested some curvature, although the non-linear term was not significant (effective degrees of freedom 1.84, *p* = 0.43). Chronotype was, therefore, categorised into five groups. A log-likelihood ratio test (LRT) showed improved model fit when adding chronotype on top of age (*p* = 0.02). Compared to an intermediate chronotype, an extremely late chronotype was associated with a 42% increased hazard of dementia (HR, 1.42 [95% CI: 1.00, 2.02]) and a slightly early chronotype with a 26% higher hazard of dementia (HR, 1.26 [95% CI: 1.08, 1.46]) (Fig. 3). Extremely early and slightly late chronotypes were not associated with increased dementia risk (Table S4 Supplementary Material).

Interaction analyses showed no significant effect modification by age (*p*-value LRT-test 0.89), educational attainment (*p*-value LRT-test 0.75) or sex (*p*-value LRT-test 0.41) (Tables S5–S7 Supplementary Material).

The first sensitivity analysis using three groups of chronotype showed that an early chronotype was associated with a 24% higher risk to develop dementia compared to an intermediate chronotype (HR, 1.24 [95% CI: 1.07, 1.43]) (Table S8 Supplementary Material). The second sensitivity analysis showed that after adjustment of sleep duration the associations between chronotype and dementia remain the same (Table S9 Supplementary Material).

## Discussion

This prospective cohort study found that individuals aged 60 years and older with a slightly early (i.e., midpoint sleep >2:30 and ≤3:30), and an extremely late chronotype (i.e., midpoint sleep >5:30) had a higher risk of dementia than those with an intermediate chronotype. These associations were not moderated by age, sex or educational attainment.

### Current evidence chronotype and dementia

Individuals aged 60 years and older with an extremely late chronotype had a 42% higher risk of dementia compared to those with an intermediate chronotype in our study. However, the confidence interval was wide, limiting the precision and making it difficult to draw firm conclusions. Previous studies examining chronotype in relation to dementia risk or cognitive decline mostly conceptualised chronotype as a personality trait (i.e., using the Morningness-Eveningness Questionnaire (MEQ), which aims to identify morning and evening persons (Horne and Ostberg, 1976)), rather than a phase of entrainment measured with the MCTQ (Roenneberg, 2015). As the MEQ and MCTQ have different aims, they are not interchangeable (Roenneberg, 2015). Previous studies have linked chronotype to cognitive outcomes (Ahn *et al.*, 2024; Cox *et al.*, 2019; Thapa *et al.*, 2020; C. Wang and Holtzman, 2020; Wenzler *et al.*, 2025). For example, being an evening person based on the MEQ was cross-sectionally associated with dementia risk based on the Mini-Mental State Examination (MMSE) score (Thapa *et al.*, 2020). Longitudinal studies using the MCTQ have shown that a later chronotype is associated with greater decline in executive functioning (Wenzler *et al.*, 2025) and increased subjective memory complains (Ahn *et al.*, 2024), although other studies, including those incorporating genetic measures of chronotype, reported no association (Cox *et al.*, 2019; Q. Wang *et al.*, 2023). In addition to the late chronotype findings, our results also suggest a potential risk among individuals with a (slightly) early chronotype. While most studies only report adverse effects for late chronotypes, some also indicate the negative cognitive outcomes for early chronotypes. Guo et al., using UK Biobank data, examined the association between chronotype and dementia, using Mendelian randomisation. They found no association between the chronotype based on PRS and dementia, but chronotype based on MCTQ was associated with all-cause dementia (Guo *et al.*, 2024). In this study chronotype was dichotomised, potentially obscuring effects in extreme groups because individuals with intermediate chronotypes were divided between categories. Our study used a more detailed categorisation of chronotype, allowing detection of effects in smaller subgroups. In line with our findings, Kim et al., also found that an earlier chronotype measured with the MCTQ, was associated with faster temporal lobe atrophy and poorer verbal learning and visual memory compared to an intermediate chronotype (Kim *et al.*, 2023). Lastly, Suh et al., reported a protective effect of a late chronotype for global cognitive decline and a higher, though non-significant, risk among early chronotypes (Suh *et al.*, 2018). Although some evidence supports associations between chronotype and cognitive outcomes, future studies should clearly distinguish between chronotype as a personality trait and as a phase of entrainment (i.e., the timing of your biological clock), as these have different clinical implications (Roenneberg, 2015). Further research should also examine the biological and behavioural mechanisms underlying these associations.

### Late chronotype and risk of dementia

The association between a late chronotype and increased dementia risk may be partially explained by biological mechanisms related to CR disruption. With ageing, the amplitude of rhythmic gene expression by the circadian clock is reduced, affecting cortisol, melatonin and body temperature regulation. At the same time, there is a gradual loss of brain cells, including those in the SCN the brains primary pacemaker (Ghorbani Shirkouhi *et al.*, 2025). Declines in gene expression and SCN cell volume can cause subtle circadian misalignment, which may impair protein expression (Verma *et al.*, 2023), weaken defences against oxidative stress (Verma *et al.*, 2023), and compromise blood–brain barrier function (Schindler *et al.*, 2025). Minor circadian disruptions can reduce process effectiveness, leaving the brain more vulnerable. Collectively, these changes increase susceptibility to cognitive dysfunction and neurodegenerative diseases. In addition to biological mechanisms, behavioural factors associated with chronotype may contribute to dementia risk. Although older adults might experience less social jetlag due to more flexible schedules (Roenneberg *et al.*, 2019), late chronotypes may have been exposed to prolonged circadian misalignment during midlife. Social jetlag occurs when fixed schedules force individuals to wake before obtaining sufficient sleep. People experiencing social jetlag are more likely to consume alcohol, smoke and have a higher body mass index, all established risk factors for cognitive decline and dementia (Livingston *et al.*, 2024). One study found that sleep quality and smoking partially mediated the relationship between a late chronotype and cognitive decline (Wenzler *et al.*, 2025). Moreover, even when sleep duration is similar, late chronotypes report higher subjective sleepiness (Zou *et al.*, 2022), which may prompt compensatory behaviours that negatively impact long-term health. Together, the combined impact of circadian misalignment, lifestyle factors and increased vulnerability may increase dementia risk.

### Early chronotype and risk of dementia

Although late chronotypes are typically associated with adverse health outcomes, our findings suggest that a slightly early chronotype may also be linked to increased dementia risk. Several pathways may explain this unexpected association. Participants with a slightly early chronotype had a sleep midpoint between 2:30 and 3:30 am, corresponding to a sleep window of approximately 22:30–23:30 to 6:30–7:30, assuming 8 hours of sleep. Although this schedule is not extremely early, evening social events such as sports classes, parties or dinners may conflict with their preferred sleep rhythm. Consequently, they may either skip such events, limiting social contacts or attend them at the cost of a reduction in either sleep duration or quality (acknowledging that sensitivity analyses found no association between sleep duration and dementia risk). Another factor may be hearing loss. Gao et al., found a suggestive association between morning chronotype and hearing loss (Gao *et al.*, 2024), which is itself a risk factor for dementia. Individuals with an extreme early chronotype were not at higher risk compared with intermediate chronotypes. However, the small sample size (2%) and wide confidence interval indicate limited precision, meaning a true association cannot be excluded. When all early chronotypes were combined in a sensitivity analysis, early chronotype was associated with higher dementia risk, likely due to increased statistical power after merging categories. Lifestyle differences may also play a role. Individuals with a slightly early chronotype may have adjusted their schedule to societal norms, whereas extremely early chronotypes may have aligned their life more closely with their biological clock, reducing negative indirect effects. Genetic differences may contribute as well. Polymorphisms in chronotype-related genes have been linked to psychiatric disease risk (Zou *et al.*, 2022). Research investigating these pathways is limited. Future studies could investigate both modifiable and non-modifiable risk factors to better understand how chronotype influences dementia risk.

### Operationalisation and categorisation of chronotype

In the current study, only 21% of the individuals had a late chronotype, with just 3% having an extremely late chronotype. This small proportion limits the health implications in older adults. By contrast, the MCTQ database with 300,000 entries worldwide, including the Netherlands, shows about 40% of individuals having a late chronotype, with 20% classified as moderately or extreme late (Roenneberg *et al.*, 2019). Our sample had less individuals in the extreme, moderate and slightly early categories, and roughly twice the proportion of intermediate chronotypes. This discrepancy raises the question of whether older adults are underrepresented in early and late chronotypes, or if traditional cut-offs are unsuitable for ageing populations. Chronotypes shift earlier with age due to SCN neuron loss, reduced rhythmic gene expression and declining sleep pressure, making late types less common and current categories less representative (Hood and Amir, 2017). Refining chronotype categories for age-specific distributions could improve study accuracy and clarify what constitutes morning or evening types in later life. Another challenge is the lack of consensus on categorising chronotype. Some studies use only early versus late groups, splitting intermediates who often have the lowest health risk (Thapa *et al.*, 2020; Guo *et al.*, 2024). Others use sample-specific cut-offs, such as median plus or minus one standard deviation. Our findings show intermediate chronotypes had the lowest dementia risk, whereas slightly early or extreme late chronotypes were at higher risk. These inconsistencies highlight the need for validated and standardised chronotype measures in health research.

### Strengths and limitations

The current study has several strengths. Chronotype was measured using the validated MCTQ, facilitating the comparison with other studies. The large sample size allowed a detailed categorisation of chronotype. Additionally, there was no loss to follow-up, as all participants who completed the MCTQ at baseline were tracked until the study’s end in 2024 using registry data. Only cognitively healthy individuals at baseline were included as participants diagnosed with dementia before baseline or during the 3–7 years between baseline and start follow-up were excluded. These strengths enhance the study’s validity, though several limitations must be acknowledged. The registry data are based on healthcare declarations, which serve as proxies rather than formal clinical dementia diagnoses. Some indicators, such as dementia-specific medication, strongly suggest a diagnosis, whereas others, including referrals to memory clinics or internal medicine for dementia-related symptoms, may also capture individuals without dementia. Follow-up was relatively short because dementia indicators from Vektis were only reliably available from 2017 onwards, resulting in a maximum of 7 years. Moreover, lag time varied between individuals, although adjusted for in the analysis. Finally, despite the large sample size, the number of participants with extreme chronotypes was limited, requiring merging of extreme early or late chronotypes with moderately early or late groups, which may reduce granularity for the extremes.

## Conclusion

To conclude, individuals aged 60 years and older with slightly early or extremely late chronotypes were at higher risk for dementia over 6.6 years. Chronotype can be assessed with short questionnaires and may serve as a low-burden risk marker complementing existing assessments. Future studies should employ tools that capture more extreme chronotypes beyond the simple early-late dichotomy, investigate whether modifiable risk factors mediate the association and replicate findings in independent cohorts. Longitudinal studies with longer follow-up (≈30 years) are needed to explore the impact of midlife chronotype on late-life dementia. Elucidating on these pathways could position chronotype as an entry point for personalised preventive strategies.

## Supporting information

10.1017/S2045796026100687.sm001 Wenzler et al. supplementary material 1 Wenzler et al. supplementary material

10.1017/S2045796026100687.sm002 Wenzler et al. supplementary material 2 Wenzler et al. supplementary material

## Data Availability

Data are not publicly available and may be obtained from third parties. Researchers can apply to access the lifelines data used in this study (project number OV23_00871) via http://www.lifelines-biobank.com. Dementia indicator and mortality data can be requested from the System of Social Statistical Databases (SSB) of Statistics Netherlands.

## Supplementary material

The supplementary material for this article can be found at https://doi.org/10.1017/S2045796026100687.

## References

[ref1] Ahn EK, Yoon K and Park JE (2024) Association between sleep hours and changes in cognitive function according to the morningness-eveningness type: A population-based study. *Journal of Affective Disorders* 345, 112–119. 10.1016/j.jad.2023.10.122 37865346

[ref2] Boivin DB, Boudreau P and Kosmadopoulos A (2022) Disturbance of the circadian system in shift work and its health impact. *Journal of Biological Rhythms* 37(1), 3–28. 10.1177/07487304211064218 34969316 PMC8832572

[ref3] Chauhan S, Norbury R, Faßbender KC, Ettinger U and Kumari V (2023) Beyond sleep: A multidimensional model of chronotype. *Neuroscience and Biobehavioral Reviews* 148, 105114. 10.1016/j.neubiorev.2023.105114 36868368

[ref4] Cox SR, Ritchie SJ, Allerhand M, Hagenaars SP, Radakovic R, Breen DP, Davies G, Riha RL, Harris SE, Starr JM and Deary IJ (2019) Sleep and cognitive aging in the eighth decade of life. *Sleep* 42(4). 10.1093/sleep/zsz019 PMC6448287 30668819

[ref5] Didikoglu A, Maharani A, Payton A, Pendleton N and Canal MM (2019) Longitudinal change of sleep timing: Association between chronotype and longevity in older adults. *Chronobiology International* 36(9), 1285–1300. 10.1080/07420528.2019.1641111 31328571

[ref6] Fischer D, Lombardi DA, Marucci-Wellman H and Roenneberg T (2017) Chronotypes in the US – Influence of age and sex. *PLoS ONE* 12(6), e0178782. 10.1371/journal.pone.0178782 28636610 PMC5479630

[ref7] Gao Y, Qiu Y and Lu S (2024) Genetically predicted sleep traits and sensorineural hearing loss: A Mendelian randomization study. *Laryngoscope* 134(11), 4723–4729. 10.1002/lary.31550 38818872

[ref8] Ghorbani Shirkouhi S, Karimi A, Khatami SS, Asgari Gashtrodkhani A, Kamari F, Blaabjerg M and Andalib S (2025) The clock and the brain: Circadian rhythm and Alzheimer’s Disease. *Current Issues in Molecular Biology* 47(7), 547. 10.3390/cimb47070547 40729016 PMC12294067

[ref9] Guo C, Harshfield EL and Markus HS (2024) Sleep characteristics and risk of stroke and dementia: An observational and Mendelian randomization study. *Neurology* 102(5), e209141. 10.1212/WNL.0000000000209141 38350061 PMC11067695

[ref10] Hood S and Amir S (2017) The aging clock: Circadian rhythms and later life. *Journal of Clinical Investigation* 127(2), 437–446. 10.1172/JCI90328 28145903 PMC5272178

[ref11] Horne JA and Ostberg O (1976) A self-assessment questionnaire to determine morningness-eveningness in human circadian rhythms. *International Journal of Chronobiology* 4(2), 97–110. 1027738

[ref12] Juda M (2010) *The Importance of Chronotype in Shift Work Research*. Inaugural dissertation (PhD), Ludwig-Maximilians-Universität.

[ref13] Kantermann T, Sung H and Burgess HJ (2015) Comparing the Morningness-Eveningness Questionnaire and Munich ChronoType Questionnaire to the dim light melatonin onset. *Journal of Biological Rhythms* 30(5), 449–453. 10.1177/0748730415597520 26243627 PMC4580371

[ref14] Kim HJ, Kim REY, Kim S, Lee SK, Lee HW and Shin C (2023) Earlier chronotype in midlife as a predictor of accelerated brain aging: A population-based longitudinal cohort study. *Sleep* 46(6). 10.1093/sleep/zsad108 37061816

[ref15] Livingston G, Huntley J, Liu KY, Costafreda SG, Selbæk G, Alladi S, Ames D, Banerjee S, Burns A, Brayne C, Fox NC, Ferri CP, Gitlin LN, Howard R, Kales HC, Kivimäki M, Larson EB, Nakasujja N, Rockwood K and Mukadam N (2024) Dementia prevention, intervention and care: 2024 report of the Lancet standing commission. *The Lancet* 404(10452), 572–628. 10.1016/S0140-6736(24)01296-0 39096926

[ref16] Moeller JS and Kriegsfeld LJ (2023) Circadian rhythms and cognitive functioning. In Fonken L. K. and Nelson R. J. (eds.), *Biological Implications of Circadian Disruption: A Modern Health Challenge*. Cambridge: Cambridge University Press, 135–164.

[ref17] Montaruli A, Castelli L, Mulè A, Scurati R, Esposito F, Galasso L and Roveda E (2021) Biological rhythm and chronotype: New perspectives in health. *Biomolecules* 11(4), 487. 10.3390/biom11040487 33804974 PMC8063933

[ref18] Musiek ES and Holtzman DM (2016) Mechanisms linking circadian clocks, sleep, and neurodegeneration. *Circadian Physiology* 354(6315), 1004–1008. http://science.sciencemag.org/10.1126/science.aah4968 PMC5219881 27885006

[ref19] Musiek ES, Xiong DD and Holtzman DM (2015) Sleep, circadian rhythms, and the pathogenesis of Alzheimer Disease. *Experimental & Molecular Medicine* 47(3), e148–e148. 10.1038/EMM.2014.121 25766617 PMC4351409

[ref20] Partonen T (2023) Extreme chronotype, regardless of misalignment, links to cardiovascular disease risks. *Sleep* 46(6). 10.1093/sleep/zsad081 36964898

[ref21] Roenenberg T, Keller LK, Fischer D, Maera JL, Vetter C and Winnebeck EC (2015) Human activity and rest in situ. *Methods in Enzymology* 552, 257–283. 25707281 10.1016/bs.mie.2014.11.028

[ref22] Roenneberg T (2015) Having trouble typing? what on earth is chronotype? *Journal of Biological Rhythms* 30(6), 487–491. 10.1177/0748730415603835 26446872

[ref23] Roenneberg T, Kuehnle T, Juda M, Kantermann T, Allebrandt K, Gordijn M and Merrow M (2007) Epidemiology of the human circadian clock. *Sleep Medicine Reviews* 11(6), 429–438. 10.1016/j.smrv.2007.07.005 17936039

[ref24] Roenneberg T, Pilz LK, Zerbini G and Winnebeck EC (2019) Chronotype and social jetlag: A (self-) critical review. *Biology* 8(3), 54. 10.3390/biology8030054 31336976 PMC6784249

[ref25] Roenneberg T, Wirz-Justice A and Merrow M (2003) Life between clocks: Daily temporal patterns of human chronotypes. *Journal of Biological Rhythms* 18(1), 80–90. 10.1177/0748730402239679 12568247

[ref26] Schindler KA, Torices S, Schurhoff N, Gallo DI and Toborek M (2025) The intersection of circadian rhythms and the blood-brain barrier with drug efficacy and delivery in neurological disorders. *Advanced Drug Delivery Reviews* 224. 10.1016/j.addr.2025.115645. PMC12321092 40614866

[ref27] Scholtens S, Smidt N, Swertz MA, Bakker SJL, Dotinga A, Vonk JM, Van Dijk F, Van Zon SKR, Wijmenga C, Wolffenbuttel BHR and Stolk RP (2015) Cohort profile: LifeLines, a three-generation cohort study and biobank. *International Journal of Epidemiology* 44(4), 1172–1180. 10.1093/ije/dyu229 25502107

[ref28] Suh SW, Han JW, Lee JR, Byun S, Kwon SJ, Oh SH, Lee KH, Han G, Hong JW, Kwak KP, Kim BJ, Kim SG, Kim JL, Kim TH, Ryu SH, Moon SW, Park JH, Seo J, Youn JC and Kim KW (2018) Sleep and cognitive decline: A prospective nondemented elderly cohort study. *Annals of Neurology* 83(3), 472–482. 10.1002/ana.25166 29394505

[ref29] Thapa N, Kim B, Yang JG, Park HJ, Jang M, Son HE, Kim GM and Park H (2020) The relationship between chronotype, physical activity and the estimated risk of dementia in community-dwelling older adults. *International Journal of Environmental Research & Public Health* 17(10), 3701. 10.3390/ijerph17103701 32456356 PMC7277473

[ref30] van den Pol H (2020) New model giving insight in group of 256,000 people living with dementia in the Netherlands. *Alzheimer’s and Dementia* 16(S10). 10.1002/alz.040854

[ref31] Verma AK, Singh S and Rizvi SI (2023) Aging, circadian disruption and neurodegeneration: Interesting interplay. *Experimental Gerontology* 172, 112076. 10.1016/j.exger.2022.112076 36574855

[ref32] Vosko AM, Colwell CS and Avidan AY (2010) Jet lag syndrome: Circadian organization, pathophysiology, and management strategies. *Nature and Science of Sleep* 2, 187–198. 10.2147/NSS.S6683 PMC3630947 23616709

[ref33] Wang C and Holtzman DM (2020) Bidirectional relationship between sleep and Alzheimer’s disease: Role of amyloid, tau, and other factors. *Neuropsychopharmacology* 45(1), 104–120. 10.1038/s41386-019-0478-5 31408876 PMC6879647

[ref34] Wang Q, Xu S, Liu F, Liu Y, Chen K, Huang L, Xu F and Liu Y (2023) Causal relationship between sleep traits and cognitive impairment: A Mendelian randomization study. *Journal of Evidence-Based Medicine* 16(4), 485–494. 10.1111/jebm.12576 38108111

[ref35] Wenzler AN, Liefbroer AC, Voshaar RCO and Smidt N (2025) Chronotype as a potential risk factor for cognitive decline: The mediating role of sleep quality and health behaviours in a 10-year follow-up study. *The Journal of Prevention of Alzheimer’s Disease* 12(6), 100168. 10.1016/j.tjpad.2025.100168 PMC12434255 40221238

[ref36] Wilking M, Ndiaye M, Mukhtar H and Ahmad N (2013) Circadian rhythm connections to oxidative stress: Implications for human health. *Antioxidants & Redox Signaling* 19(2), 192–208. 10.1089/ars.2012.4889 23198849 PMC3689169

[ref37] Zavada A, Gordijn MC, Beersma DG, Daan S and Roenneberg T (2005) Comparison of the Munich Chronotype Questionnaire with the Horne‐Östberg’s Morningness‐Eveningness score. *The Journal of Biological and Medical Rhythm Research* 22(2), 267–278. 10.1081/CBI-200053536 16021843

[ref38] Zou H, Zhou H, Yan R, Yao Z and Lu Q (2022) Chronotype, circadian rhythm, and psychiatric disorders: Recent evidence and potential mechanisms. *Frontiers in Neuroscience.* 16. 10.3389/fnins.2022.811771. PMC9399511 36033630

