# Supplementary material for: The association between chronotype and incident dementia: exploring age, educational-attainment and sex differences
Source: Epidemiol Psychiatr Sci. 2026 May 7;35:e31. doi: 10.1017/S2045796026100687 (PMC13150776; doi:10.1017/S2045796026100687)
Supplement: Wenzler et al. supplementary material 2 — Wenzler et al. supplementary material [file S2045796026100687sup002.docx]

**Supplementary Matter**

**The Association Between Chronotype and incident Dementia: Exploring Age, Educational-Attainment, and Sex Differences**

A.N. Wenzler ^1^, A.C. Liefbroer ^1,2,3^, R.C. Oude Voshaar ^3^, N. Smidt ^1,4^

^1^ Department of Epidemiology, University of Groningen, University Medical Center Groningen, Groningen, The Netherlands

^2^ Netherlands Interdisciplinary Demographic Institute (NIDI)–Royal Netherlands Academy of Sciences (KNAW), Lange Houtstraat 19, 2511 CV, The Hague, The Netherlands

^3^ Department of Sociology, Vrije Universiteit Amsterdam (VU), Amsterdam, The Netherlands

^4^ Department of Psychiatry, University of Groningen, University Medical Center Groningen, Groningen, The Netherlands.

Corresponding author: Ana Wenzler, University Medical Center Groningen, University of Groningen, Department of Epidemiology, FA40 PO30.001, Groningen, 9700 RB, The Netherlands, [a.n.wenzler@umcg.nl](mailto:a.n.wenzler@umcg.nl)

ORCID

Ana N. Wenzler- <https://orcid.org/0009-0005-2781-8595>

Richard C Oude Voshaar- <https://orcid.org/0000-0003-1501-4774>

Aart C Liefbroer- <https://orcid.org/0000-0002-7884-3150>

Nynke Smidt- <https://orcid.org/0000-0002-2778-8841>

Table of content

[Figure 1 3](#_Toc225330436)

[Table 1 4](#_Toc225330437)

[Table 2 6](#_Toc225330438)

[Table 3 7](#_Toc225330439)

[Figure 2 8](#_Toc225330440)

[Table 4 9](#_Toc225330441)

[Table 5 10](#_Toc225330442)

[Table 6 11](#_Toc225330443)

[Table 7 13](#_Toc225330444)

[Table 8 14](#_Toc225330445)

[Table 9 15](#_Toc225330446)

[References 16](#_Toc225330447)

# Figure 1

This Figure presents the timeline of the current study design. From 2006 to 2017 several dementia indicators have been added to the Vektis dementia indicator dataset (Supplementary Matter 2). From 2017 dementia could be determined by the combination of the then available indicators. Therefore, from 2018 onwards incident dementia could be predicted. Chronotype was measured by the MCTQ among Lifelines participants between 2011 and 2015. As the follow-up of dementia started in 2018, there was a lag time between the measurement of chronotype and dementia follow-up. This lag time differed between 3 and 7 years, depending on when MCTQ data were provided. The end of follow-up was in 2024.


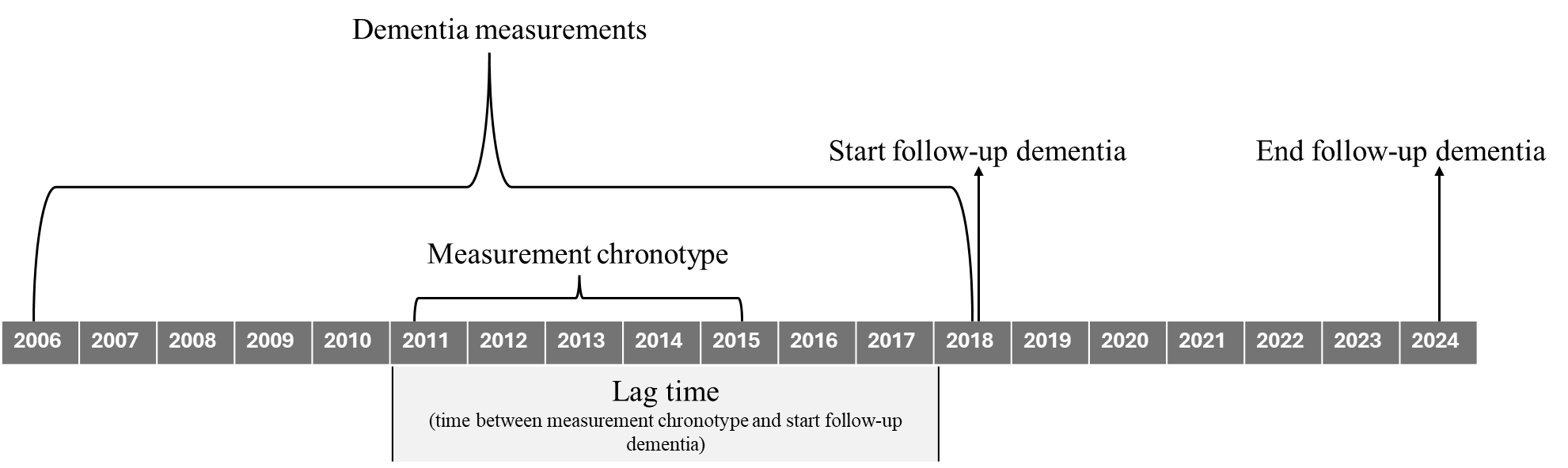


Figure 1 Study timeline, including timing of measurement of exposure, outcome, and start of follow-up

# Table 1

Table with the overview of available dementia indicators over the years. Below the table explanation of the different dementia indicators is presented.

Table 1 Years of collection dementia indicators by Vektis

| CBS Flag (code) | 2006 | 2007 | 2008 | 2009 | 2010 | 2011 | 2012 | 2013 | 2014 | 2015 | 2016 | 2017 | 2018 | 2019 | 2020 | 2021 | 2022 | 2023 | 2024 | 2025 |
| --- | --- | --- | --- | --- | --- | --- | --- | --- | --- | --- | --- | --- | --- | --- | --- | --- | --- | --- | --- | --- |
| F |  |  |  |  |  |  |  |  |  |  |  |  |  |  |  |  |  |  |  |  |
| MSZ (neurology) |  |  |  |  |  |  |  |  |  |  |  |  |  |  |  |  |  |  |  |  |
| MSZ (geriatrics) |  |  |  |  |  |  |  |  |  |  |  |  |  |  |  |  |  |  |  |  |
| GGZ |  |  |  |  |  |  |  |  |  |  |  |  |  |  |  |  |  |  |  |  |
| ZIN Wlz (stay) |  |  |  |  |  |  |  |  |  |  |  |  |  |  |  |  |  |  |  |  |
| ZIN Wlz (VPT) |  |  |  |  |  |  |  |  |  |  |  |  |  |  |  |  |  |  |  |  |
| PGB Wlz |  |  |  |  |  |  |  |  |  |  |  |  |  |  |  |  |  |  |  |  |
| ZIN Wlz (function) |  |  |  |  |  |  |  |  |  |  |  |  |  |  |  |  |  |  |  |  |
| Wlz indication |  |  |  |  |  |  |  |  |  |  |  |  |  |  |  |  |  |  |  |  |
| MSZ (internal med.) |  |  |  |  |  |  |  |  |  |  |  |  |  |  |  |  |  |  |  |  |
| WV |  |  |  |  |  |  |  |  |  |  |  |  |  |  |  |  |  |  |  |  |
| Wlz indication |  |  |  |  |  |  |  |  |  |  |  |  |  |  |  |  |  |  |  |  |

F

Dementia-related extramural medication is available since 2006. This is a single aggregated category including the following medication: Rivastigmine, Donepezil, Memantine, Galantamine.

MSZ

All dementia-related hospital diagnosis were assessed separately per department. Dementia syndromes at the department of neurology were included since 2007. Memory problems and dementia at the Department of Clinical Geriatrics were included since 2007. Memory problems and dementia at the Department of Internal Medicine were included since 2014.

GGZ

Dementia-related diagnoses in mental health care (available since 2007). This category encompasses diagnoses of delirium, dementia, amnestic disorders, and other cognitive impairments.

Wlz declarations (ZIN Wlz and PGB Wlz)

WLZ refers to *Wet Langdurige Zorg* (Dutch Long-Term Care Act), which provides access to intensive, long-term care for individuals with chronic physical or mental conditions, including dementia. All Wlz-related claims were assessed separately between 2012 and 2017. Wlz Residential Care (verblijf) was included with incication ZZP VV5 and performance codes Z051 and Z053. Wlz Fully Package at Home (VPT) was included with indication VV5 and performance codes V051 and V053. Wlz Personal budget (PGB) was included with indication VV5 and psychogeriatric basis. Wlz services (function) was included as having day on psychogeriatric basis and performance code H533. All WLZ declarations have been combined into one variable from 2018 onwards.

WV

Dementia-related community nursing is available since 2017. This category comprises a collection of performance cores related to community nursing for individuals with dementia. The following is included: 1) Care for frail older adults and chronically ill patients lasting longer than three months, with a psychogeriatric of psychiatric indication, 2) dementia care as part of integrated care pathways, 3) psychogeriatric care, 4) complex psychogeriatric care (PG-complex) with dementia-specific performance codes.

Wlz indication

This concerns one category with information on the first or second ground for psychogeriatric care (PG). This variable contains all previous WLz declarations gathered from 2012 to 2017.

# Table 2

Table 2 Association between age and dementia risk

| Variable | Study population  n = 16,757 | p-value ^$^ |
| --- | --- | --- |
|  | HR (95% CI) |  |
| Age ^*^ | 1.18 (1.16, 1.20) | **<0.001** |
| Age squared ^*^ | 1.00 (1.00, 1.00) ^^^ | **<0.001** |
| Lag time ^#^ | 1.04 (0.97, 1.11) | 0.32 |
| ^*^ Age was included as years  ^#^ The time between filling in the MCTQ and the start of follow-up was referred to as lag time and differed from 3 to 7 years  ^$^ A p-value of 0.05 or lower was deemed statistically significant  ^^^ The HR for age-squared was below 1.00. The upper and lower 95% for the HR for age-squared was below 1.00. Due to rounding, a HR of 1.00 including 95% CI 1.00, 1.00 is presented. | | |

# Table 3

Table 3 Association between continuous chronotype and dementia

| Variable | Study population  n = 16,757 | p-value ^^^ |
| --- | --- | --- |
|  | HR (95% CI) |  |
| Chronotype ^*^ | 0.95 (0.88, 1.04) | 0.24 |
| Sex (male) | 1.06 (0.94, 1.20) | 0.32 |
| Age ^#^ | 1.18 (1.16, 1.20) | **<0.001** |
| Age squared ^#^ | 1.00 (1.00, 1.00) | **<0.001** |
| Lag time ^$^ | 1.04 (0.97, 1.11) | 0.31 |
| ^*^ Chronotype was assessed at baseline with the Munich ChronoType Questionnaire (MCTQ) and was included as a continuous score of the hour of Mid-point Sleep on Free days corrected for sleep debt on workdays (MSF_SC_) (Roenneberg et al., 2003)  ^#^ Age was included as years  ^$^ The time between filling in the MCTQ and the start of follow-up was referred to as lag time and differed from 3 to 7 years  ^^^ A p-value of 0.05 or lower was deemed statistically significant | | |

# Figure 2


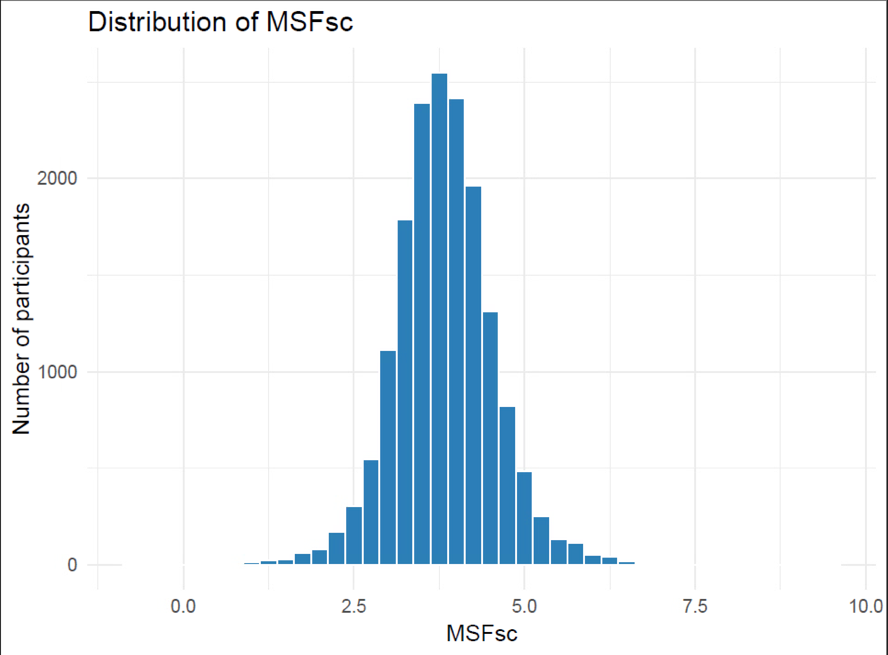


Figure 2 The distribution of chronotype in the population for analysis

# Table 4

Table 4 Association between chronotype (in five categories) and dementia

| Variable | Study population  n = 16,757 | p-value ^^^ |
| --- | --- | --- |
|  | HR (95% CI) |  |
| Chronotype – extreme early ^*^ (n = 345) | 1.07 (0.71, 1.63) | 0.74 |
| Chronotype – slight early ^*^ (n = 3,319) | 1.26 (1.08, 1.46) | **<0.001** |
| Chronotype – intermediate ^*^ (n = 9,434) | ref | ref |
| Chronotype – slight late ^*^ (n = 3,210) | 1.01 (0.86, 1.20) | 0.86 |
| Chronotype – extreme late (n = 449) | 1.42 (1.00, 2.02) | **0.05** |
| Age ^#^ | 1.18 (1.16, 1.20) | **<0.001** |
| Age squared ^#^ | 1.00 (1.00, 1.00) | **<0.001** |
| Sex (male) | 1.07 (0.95, 1.20) | 0.29 |
| Lag time ^$^ | 1.04 (0.97, 1.11) | 0.32 |
| ^*^ Individuals were categorised in five following categories: extremely early (MSF_sc_  ≤2:30), slight early (MSF_sc_ >2:00 and ≤3:30), intermediate (MSF_sc_ >3:30 and ≤4:30), late (MSF_sc_ >4:30 and ≤5:30), extreme late (MSF_sc_ >5:30)  ^#^ Age was included as years  ^$^ The time between filling in the MCTQ and the start of follow-up was referred to as lag time and differed from 3 to 7 years  ^^^ A p-value of 0.05 or lower was deemed statistically significant | | |

# Table 5

Table 5 Association between chronotype and dementia risk, including interaction term for age

| Variable | Study population  n = 16,757 | p-value ^^^ |
| --- | --- | --- |
|  | HR (95% CI) |  |
| Chronotype – extreme early ^*^ (n = 345) | 0.99 (0.57, 1.70) | 0.96 |
| Chronotype – slight early ^*^ (n = 3,319) | 1.24 (1.03, 1.50) | **0.02** |
| Chronotype – intermediate ^*^ (n = 9,434) | ref | ref |
| Chronotype – slight late ^*^ (n = 3,210) | 0.97 (0.78, 1.19) | 0.74 |
| Chronotype – extreme late (n = 449) | 1.56 (1.02, 2.38) | **0.04** |
| Age ^#^ | 1.17 (1.15, 1.20) | **<0.001** |
| Age squared ^#^ | 1.00 (1.00, 1.00) | **0.01** |
| Sex (male) | 1.07 (0.95, 1.20) | 0.29 |
| Lag time ^$^ | 1.04 (0.97, 1.11) | 0.32 |
| Age * Chronotype – extreme early | 0.95 (0.85, 1.07) | 0.41 |
| Age * Chronotype – moderate early | 1.00 (0.95, 1.04) | 0.85 |
| Age * Chronotype – intermediate | ref | ref |
| Age * Chronotype – moderate late | 1.03 (0.98, 1.09) | 0.27 |
| Age * Chronotype – extreme late | 1.00 (0.90, 1.12) | 0.94 |
| Age squared * Chronotype – extreme early | 1.01 (1.00, 1.01) | 0.28 |
| Age squared * Chronotype – moderate early | 1.00 (1.00, 1.00) | 0.76 |
| Age squared * Chronotype – intermediate | ref | ref |
| Age squared * Chronotype – moderate late | 1.00 (0.99, 1.00) | 0.46 |
| Age squared * Chronotype – extreme late | 1.00 (0.99, 1.01) | 0.55 |
| ^*^ Individuals were categorised in five following categories: extremely early (MSF_sc_  ≤2:30), slight early (MSF_sc_ >2:30 and ≤3:30), intermediate (MSF_sc_ >3:30 and ≤4:30), late (MSF_sc_ >4:30 and ≤5:30), extreme late (MSF_sc_ >5:30)  ^#^ Age was included as years  ^$^ The time between filling in the MCTQ and the start of follow-up was referred to as lag time and differed from 3 to 7 years  ^^^ A p-value of 0.05 or lower was deemed statistically significant | | |

There was no effect modification of age and age^2 on the association between chronotype and dementia risk. The LRT-test, comparing the model with and without the interaction showed a p-value of 0.89, indicating that adding the interaction to the model did not improve the model.

# Table 6

Table 6 Association between chronotype and dementia risk, including interaction term for educational attainment

| Variable | Study population  n = 16,757 | p-value ^^^ |
| --- | --- | --- |
|  | HR (95% CI) |  |
| Chronotype – extreme early ^*^ (n = 345) | 1.01 (0.58, 1.75) | 0.98 |
| Chronotype – slight early ^*^ (n = 3,319) | 1.24 (1.01, 1.51) | **0.04** |
| Chronotype – intermediate ^*^ (n = 9,434) | ref | ref |
| Chronotype – slight late ^*^ (n = 3,210) | 1.03 (0.82, 1.29) | 0.78 |
| Chronotype – extreme late (n = 449) | 1.19 (0.72, 1.96) | 0.50 |
| Age ^#^ | 1.17 (1.15, 1.20) | **<0.001** |
| Age squared ^#^ | 1.00 (1.00, 1.00) | **<0.001** |
| Low educational attainment | ref | ref |
| Middle educational attainment | 0.95 (0.76, 1.17) | 0.61 |
| High educational attainment | 0.96 (0.79, 1.17) | 0.73 |
| Sex (male) | 1.08 (0.96, 1.23) | 0.20 |
| Lag time ^$^ | 1.04 (0.97, 1.12) | 0.28 |
| Low educational attainment * Chronotype – extreme early | ref | ref |
| Low educational attainment * Chronotype – moderate early | ref | ref |
| Low educational attainment * Chronotype – intermediate | ref | ref |
| Low educational attainment * Chronotype – moderate late | ref | ref |
| Low educational attainment * Chronotype – extreme late | ref | ref |
| Middle educational attainment * Chronotype – extreme early | 1.28 (0.45, 3.67) | 0.65 |
| Middle educational attainment * Chronotype – moderate early | 0.91 (0.61, 1.35) | 0.64 |
| Middle educational attainment * Chronotype – intermediate | ref | ref |
| Middle educational attainment * Chronotype – moderate late | 0.84 (0.54, 1.31) | 0.44 |
| Middle educational attainment * Chronotype – extreme late | 2.18 (0.96, 4.95) | 0.06 |
| High educational attainment * Chronotype – extreme early | 1.01 (0.32, 3.15) | 0.99 |
| High educational attainment * Chronotype – moderate early | 1.09 (0.75, 1.58) | 0.66 |
| High educational attainment * Chronotype – intermediate | ref | ref |
| High educational attainment * Chronotype – moderate late | 1.08 (0.73, 1.61) | 0.70 |
| High educational attainment * Chronotype – extreme late | 0.97 (0.37, 2.54) | 0.95 |
| ^*^ Individuals were categorised in five following categories: extremely early (MSF_sc_  ≤2:30), slight early (MSF_sc_ >2:00 and ≤3:30), intermediate (MSF_sc_ >3:30 and ≤4:30), late (MSF_sc_ >4:30 and ≤5:30), extreme late (MSF_sc_ >5:30)  ^#^ Age was included as years  ^$^ The time between filling in the MCTQ and the start of follow-up was referred to as lag time and differed from 3 to 7 years  ^^^ A p-value of 0.05 or lower was deemed statistically significant | | |

There was no effect modification of educational attainment on the association between chronotype and dementia risk. The LRT-test, comparing the model with and without the interaction showed a p-value of 0.75, indicating that adding the interaction to the model did not improve the model.

# Table 7

Table 7 Association between chronotype and dementia risk, including interaction term for age

| Variable | Study population  n = 16,757 | p-value ^^^ |
| --- | --- | --- |
|  | HR (95% CI) |  |
| Chronotype – extreme early ^*^ (n = 345) | 0.67 (0.33, 1.36) | 0.85 |
| Chronotype – slight early ^*^ (n = 3,319) | 1.22 (0.99, 1.50) | 0.07 |
| Chronotype – intermediate ^*^ (n = 9,434) | ref | ref |
| Chronotype – slight late ^*^ (n = 3,210) | 0.98 (0.78, 1.22) | 0.86 |
| Chronotype – extreme late (n = 449) | 1.36 (0.88, 2.10) | 0.16 |
| Age ^#^ | 1.18 (1.16, 1.20) | **<0.001** |
| Age squared ^#^ | 1.00 (1.00, 1.00) | **<0.001** |
| Sex (male) | 1.02 (0.86, 1.19) | 0.85 |
| Lag time ^$^ | 1.04 (0.97, 1.11) | 0.32 |
| Sex * Chronotype – extreme early | 2.34 (0.98, 5.61) | 0.06 |
| Sex * Chronotype – moderate early | 1.07 (0.80, 1.44) | 0.66 |
| Sex * Chronotype – intermediate | ref | ref |
| Sex * Chronotype – moderate late | 1.08 (0.77, 1.50) | 0.66 |
| Sex * Chronotype – extreme late | 1.11 (0.53, 2.33) | 0.78 |
| ^*^ Individuals were categorised in five following categories: extremely early (MSF_sc_  ≤2:30), slight early (MSF_sc_ >2:00 and ≤3:30), intermediate (MSF_sc_ >3:30 and ≤4:30), late (MSF_sc_ >4:30 and ≤5:30), extreme late (MSF_sc_ >5:30)  ^#^ Age was included as years  ^$^ The time between filling in the MCTQ and the start of follow-up was referred to as lag time and differed from 3 to 7 years  ^^^ A p-value of 0.05 or lower was deemed statistically significant | | |

There was no effect modification of sex on the association between chronotype and dementia risk. The LRT-test, comparing the model with and without the interaction showed a p-value of 0.41, indicating that adding the interaction to the model did not improve the model.

# Table 8

Table 8 Association between chronotype (three categories) and dementia

| Variable | Study population  n = 16,757 | p-value ^^^ |
| --- | --- | --- |
|  | HR (95% CI) |  |
| Chronotype –early ^*^ (n = 3,664) | 1.24 (1.07, 1.43) | **<0.001** |
| Chronotype – intermediate ^*^ (n = 9,434) | Ref | ref |
| Chronotype –late (n = 3,659) | 1.06 (0.91, 1.25) | 0.47 |
| Age ^#^ | 1.18 (1.16, 1.20) | **<0.001** |
| Age squared ^#^ | 1.00 (1.00, 1.00) | **<0.001** |
| Sex (male) | 1.06 (0.94, 1.20) | 0.29 |
| Lag time ^$^ | 1.04 (0.97, 1.11) | 0.32 |
| ^*^ Individuals were categorised in the three following categories: early chronotype (MSF_sc_ ≤3:30), intermediate chronotype (MSF_sc_ > 3:30 & ≤4:30) and late chronotype (MSF_sc_ > 4:30).  ^#^ Age was included as years  ^$^ The time between filling in the MCTQ and the start of follow-up was referred to as lag time and differed from 3 to 7 years  ^^^ A p-value of 0.05 or lower was deemed statistically significant | | |

# Table 9

Table 9 Association between chronotype (five categories) and dementia, adjusted for hours of sleep

| Variable | Study population  n = 16,757 | p-value ^^^ |
| --- | --- | --- |
|  | HR (95% CI) |  |
| Chronotype – extreme early ^*^ (n = 345) | 0.94 (0.60, 1.49) | 0.81 |
| Chronotype – slight early ^*^ (n = 3,319) | 1.29 (1.11, 1.50) | **<0.001** |
| Chronotype – intermediate ^*^ (n = 9,434) | ref | ref |
| Chronotype – slight late ^*^ (n = 3,210) | 1.02 (0.87, 1.21) | 0.80 |
| Chronotype – extreme late (n = 449) | 1.51 (1.06, 2.14) | **0.02** |
| Age ^#^ | 1.18 (1.15, 1.20) | **<0.001** |
| Age squared ^#^ | 1.00 (1.00, 1.00) | **<0.001** |
| Hours of sleep (centered) | 1.05 (0.99, 1.11) | 0.11 |
| Sex (male) | 1.05 (0.93, 1.19) | 0.40 |
| Lag time ^$^ | 1.03 (0.96, 1.10) | 0.40 |
| ^*^ Individuals were categorised in five following categories: extremely early (MSF_sc_  ≤2:30), slight early (MSF_sc_ >2:00 and ≤3:30), intermediate (MSF_sc_ >3:30 and ≤4:30), late (MSF_sc_ >4:30 and ≤5:30), extreme late (MSF_sc_ >5:30)  ^#^ Age was included as years  ^$^ The time between filling in the MCTQ and the start of follow-up was referred to as lag time and differed from 3 to 7 years  ^^^ A p-value of 0.05 or lower was deemed statistically significant | | |

# References

Roenneberg, T., Wirz-Justice, A., & Merrow, M. (2003). Life between clocks: Daily temporal patterns of human chronotypes. *Journal of Biological Rhythms*, *18*(1), 80–90. https://doi.org/10.1177/0748730402239679
